# Outcomes of metachronous para-aortic lymphadenectomy in colorectal cancer: a systematic review of the literature

**DOI:** 10.1007/s00423-023-03185-9

**Published:** 2023-12-13

**Authors:** Oluwatobi O. Onafowokan, Jennifer Redfern, Agastya Patel, Thomas Satyadas, Minas Baltatzis

**Affiliations:** 1 Department of Hepato-Pancreato-Biliary Surgery, Manchester Royal Infirmary, Manchester University Foundation Trust, Oxford Rd, Manchester, M13 9WL UK; 2 Department of Upper GI Surgery, Salford Royal Foundation Trust, Northern Care Alliance, Stott Ln, Salford, M6 8HD UK

**Keywords:** Lymph node excision, Para-aortic lymph node, Metastasis, Metachronous, Colorectal neoplasms

## Abstract

**Introduction and aim:**

Para-aortic lymph node metastasis associated with colorectal cancer is a very rare occurrence, but at the same time an important predictor of survival. Despite its importance, there is still no gold standard management strategy, particularly for lymph nodes detected during follow-up, after resection of the primary tumour. Therefore, this review was undertaken to examine the evidence available on the surgical and non-surgical management of metachronous para-aortic lymph node metastasis (m-PALNM) in colorectal cancer treatment.

**Methods:**

This is a systematic review using the patient, intervention, comparison, outcome and study strategy. The literature search was undertaken using Cochrane, MEDLINE, EMBASE and PubMed databases with the following MeSH terms: lymph node excision, para-aortic lymph node, metastasis, metachronous and colorectal neoplasms.

**Results:**

Five original papers met the study criteria including 188 patients in total (55.3% male, 44.7% female). Surgical resection of the m-PALND was the management of choice in 64% of patients. Reporting styles on survival outcomes were heterogeneous. However, patients undergoing surgical management for m-PALNM had longer disease-free survival and overall survival rates.

**Conclusion:**

There is significant paucity in the evidence available on the management of m-PALNM. However, the evidence reported by this review suggests that surgical management should be considered whenever possible, with the aim of prolonging survival. Future randomised trials are needed in order to provide further high-level evidence on m-PALNM management.

## Introduction

Para-aortic lymph node metastasis (PALNM) associated with colorectal cancer (CRC), unlike liver and lung metastasis, is a rare occurrence with incidence rates reported as < 2% [1]. Despite this uncommon occurrence, PALNM is amongst the paramount prognostic factors for CRC [2].

There is still no clearly defined gold standard management strategy for PALNM. A significant contributing factor to this dilemma is the variation in definition of PALNM. According to the Japanese Society for Cancer of the Colon and Rectum PALNM is classified as regional or stage III disease [1, 3]. On the contrary, the American Joint Committee on Cancer defined PALNM as disseminated stage IV disease and the TNM staging system as M1 disease [1, 4]. This discrepancy unavoidably causes inconsistency in reporting and measuring management outcomes.

PALNM may occur synchronously or metachronously, with synchronous PALNM (s-PALNM) resulting in more advanced disease at diagnosis, and poorer patient survival outcomes, when compared with metachronous PALNM (m-PALNM) [5]. The lack of unambiguously clear distinctions between the two PALNM types in published reports [2, 6, 7], is also another contributing factor to the ambiguity of an optimal management strategy for PALNM in CRC.

Resection of synchronous and metachronous CRC-associated hepatic or pulmonary metastases is now widely practised after significant evidence of improved survival and morbidity outcomes has been presented [8, 9]. However, there is relative paucity in the literature available for the surgical and non-surgical management of PALNM in CRC, with a distinct lack of published randomised controlled trials, and the predominant evidence originating from retrospective studies of which a significant amount are case reports. Several studies report the outcomes of simultaneous resection of s-PALNM and the primary CRC [10–16]. Although the evidence supporting this approach still remains weak, the most difficult dilemma refers to the management of m-PALNM, as resection of metachronous disease requires a second laparotomy (or laparoscopy), which could increase patients’ morbidity and mortality for a questionable benefit. The aim of this study is to review and summarise the outcomes of surgical and non-surgical management of metachronous PALNM in CRC and define the pathways for further research in this field.

## Materials and methods

### Eligibility criteria

Eligibility criteria were determined using the population, intervention, comparison, outcome and study design (PICOS) strategy [17]. The relevant population included patients who had a diagnosis of isolated metachronous para-aortic lymph node metastasis (m-PALNM) on imaging (computed tomography and/or magnetic resonance imaging and/or positron emission tomography). Surgical management or resection was the intervention of interest. The comparison population were patients who underwent non-surgical management. The outcomes included, but were not limited to, disease-free survival, overall survival, histological characteristics and times to recurrence. Articles which included data on both m- and s-PALNM or other metastatic sites were only included if the m-PALNM-related data could be isolated from all others. Study designs included were randomised control trials, non-randomised trials, case–control studies, cohort studies (Levels I–III evidence). Levels of evidence were delineated using the Centre for Evidence-Based Medicine’s criteria for levels of evidence for therapeutic studies. (“Appendix 1”) [18, 19].

Papers containing non-published series, papers not providing original data, papers not written in English, case series, case reports and expert opinions (levels IV and V evidence) were excluded. Other exclusion criteria were: editorials, letters, non-human studies, cadaveric studies and articles in which PALNM occurred concurrently with distant metastases.

### Information sources

A literature search using Cochrane, MEDLINE, EMBASE and PubMed databases was conducted independently by one reviewer (OO) using MeSH terms: lymph node excision, para-aortic lymph node, metastasis, metachronous and colorectal neoplasms (“Appendix 2”). Studies were only included if published from the year 2000 and onwards. Bibliographies of relevant studies were searched for additional papers which met the inclusion criteria. The search strategy is summarised in the PRISMA flowchart (“Appendix 2”).

### Definitions

Para-aortic lymph nodes (PALNs) were defined as lymph nodes surrounding the abdominal aorta and inferior vena cava, which were located in the area from the uppermost part of the origin of the celiac trunk to the lower margin of the aortic bifurcation [3].

### Data extraction

Data were extracted on the following categories: study type, participant demographics, diagnostic modalities utilised, resection margins, primary tumour locations, tumour histology, additional therapeutic modalities utilised, surgery-related morbidity and mortality, overall survival, disease or recurrence-free survival/ interval, recurrence rates and recurrence sites.

### Assessment of risk of bias

The risk of bias assessment was performed using the National Heart, Lung and Blood Institute’s (NIH) Quality Assessment Tool [20]. The tool assesses quality based on 14 questions and provides an overall quality assessment of good, fair or poor.

### Synthesis of results

Data were extracted to populate a pre-defined series of tables addressing the clinical characteristics described above.

## Ethics review

The protocol was reviewed by the research and development department of Central Manchester University Hospitals NHS Foundation Trust who stated that as there is no direct clinical contact, and therefore further ethics committee review was not required.

## Results

### Search results

The search strategy provided a total of 1897 results, which were critically reviewed for eligibility of inclusion (“Appendix 1”). A final total of 5 papers met the study criteria, with two studies originating from South Korea, and one each from Canada, France and the USA (Table 1). There were a total of 188 participants (55.3% male, 44.7% female). Surgical management for metachronous para-aortic lymph node metastasis occurred in 64% of patients (121/188), compared with non-surgical management in 36% (67/188). The location of the primary tumour was the colon in 70% of patients, and the rectum in 30%. (Table 2).

**Table 1:** Demographics

First author/year	Country	Recruitment period	Number of patients with isolated m-PALNM (*n*)	Surgical management (*n*,%)	Non-surgical management (*n*,%)	Age	Gender (M/F)
Shibata et al. 2002 [24]	USA	1988 – 1999	25	20 (80%)	5 (20%)	Median 55	13/12
Min et al. 2008 [22]	South Korea	1992 – 2004	38	6 (16%)	32 (84%)	Mean: 54.6 (range: 15–87)	23/15
Dumont et al. 2012 [25]	France	1988 – 2008	31	31 (100%)	0	Median: 52 (range: 42–62)	16/15
Razik et al. 2014 30]	Canada	1999 – 2010	48	48 (100%)	0	Median: 60 (range: 36–80)	22/26
Kim et al. 2020 [23]	South Korea	2004 – 2014	46	16 (35%)	30 (65%)	Mean: 57.5 (SD ± 11.1)	30/16
Total	n/a	1988 – 2014	188	121 (64%)	67 (36%)	n/a	104/84

**Table 2:** Diagnosis, disease profile and surgery

First author/year	Diagnostic workup	Location of primary (*n*) (colon/ rectum)	Cases abandoned intra-operatively	Surgical approach (open/ laparoscopic)	Morbidity (%)	Mortality (%)
Shibata et al. 2002	CT, CEA	19/6	5	20/0	28%	4%
Min et al. 2008	CT, CEA	21/17	0	6/0	33%	0
Dumont et al. 2012	CT, CEA	28/3	0	31/0	Not reported	0
Razik et al. 2014	CT, MRI, CEA	43/5	0	48/0	52%	0
Kim et al. 2020	CT, MRI, PET- CT, CEA	20/26	Not reported	16/0	Not reported	0
Total	n/a	131/57	5	121/0	40%	0.8%

### Surgery and short-term outcomes

Of the 121 patients who underwent surgical resection of m-PALNM, 100% underwent an open surgical approach (Table 2). Resection margins were R0 in 88% of patients and R1/R2 in 12% of patients. Five cases were abandoned intraoperatively (Table 3). There was a lack of consistent reporting on the amount of resected and/or positive lymph nodes. The complication rate was 40% within 30 days of surgery, including all grades of complications according to the Clavien-Dindo classification system [21]. Overall reported mortality was 0.8% within 30 days of surgery (Table 2).

**Table 3:** Histology

First author/year	Surgical resection margin	Number of resected lymph nodes (mean)	Number of positive lymph nodes (mean)	Size of largest positive lymph node (cm)
Shibata et al. 2002	R0: 75%, R1/R2: 25%	Not reported	Not reported	> 5
Min et al. 2008	R0: 100%	Not reported	Not reported	Not reported
Dumont et al. 2012	R0: 100%	12	6	> 5
Razik et al. 2014	R0: 83%, R2: 13%, R2: 4%	Not reported	Not reported	14.5
Kim et al. 2020	Not reported	16	13	2
Total	R0:88%, R1/R2:12%	n/a	n/a	n/a

### Neoadjuvant and adjuvant treatment

Neoadjuvant and adjuvant therapeutic interventions involved chemotherapy (CT), radiotherapy (RT) or a combination of both. CT and RT use was variable amongst the studies (Table 4). CT regimens were also variable, which reflects the long study period of this systematic review.

**Table 4:** Chemotherapy and radiotherapy

First author/year	Chemotherapy			Radiotherapy	
	Neoadjuvant	Adjuvant	Regimen	Neoadjuvant	Adjuvant
Shibata et al. 2002	24%	70%	Not reported	16%	0
Min et al. 2008	0	100%	5-fluorouracil-leucovorin + oxaliplatin or irinotecan	0	50%
Dumont et al. 2012	77%	74%	5-fluorouracil and leucovorin + oxaliplatin + irinotecan + cetuximab and/or bevacizumab	19%	39%
Razik et al. 2014	42%	25%	Not reported	38%	0
Kim et al. 2020	33%	94%	FOLFOX or FOLFIRI + / − Capecitabine + / − Pembrolizumab + / − Bevacizumab	0	22%

### Survival

Reporting styles on survival characteristics were heterogeneous. Patients undergoing surgical management for m-PALNM had longer disease-free survival (DFS) and overall survival (OS) rates. Three of the studies compared survival between surgical and non-surgical cohorts. (Table 5) Min et al. reported DFS and OS between resected and non-resected groups of 22 vs 18 months (mean, *p* = 0.049) and 42.5 vs 18.7 months (mean, *p* = 0.034) respectively [22]. Kim et al. reported DFS and OS rates between resected and non-resected groups of 24.4 months vs 21.6 months (mean, *p* = 0.603) and 77 vs 62 months (median, *p* = 0.055) respectively.[23]Shibata et al. reported 2-year and 5-year OS rates for resected vs non-resected groups of 60% vs 20% and 15% vs 0% respectively [24]. Time to m-PALNM is displayed in Table 6.

**Table 5:** Survival

First author/year	Disease-free survival			Overall survival		
	No resection	Resection	*p*value	No resection	Resection	*p *value
Shibata et al. 2002	Not reported	Not reported		2-year survival: 20% 5-year survival: 0%	2-year survival: 60% 5-year survival: 15%	
Min et al. 2008	18 months (mean)	22 months (mean)	0.049	18.7 months (mean)	42.5 months (mean)	0.034
Dumont et al. 2012	n/a	15 months (3–44) (median, range) 3-year disease free survival: 19%		n/a	49 months (median) {no range reported} 3-year survival: 71%	
Razik et al. 2014	n/a	5-year disease free survival: 46%		n/a	80 months median 5-year survival: 70%	
Kim et al. 2020	21.6 months ± 17.9 (mean ± SD)	24.4 months ± 12.5 (mean ± SD)	0.603	62 months (median)	77 months (median)	0.055

**Table 6:** Time to recurrence

Author/year	Time to recurrence—mean (months)	Time to recurrence—median (months)	m-PALNM presentation	Recurrence post-PALND
Shibata 2002	Not reported	23	56% symptoms (not specified), 20% elevated CEA, 24% routine CT scan	“Local recurrence” (92%), “distant disease” (8%)
Min et al. 2008	19.9	14	63.2% elevated CEA, 36.8% on routine CT scan	Liver (94.7%), lung (81.6%), brain (7.9%), bone (28.9%)
Dumont 2012	Not reported	Not reported	36% elevated CEA	Lung (40%), liver (31%), peritoneum (10%), mediastinal LN (16%), suprarenal PALN (26%), infrarenal PALN (13%), iliac LN (10%)
Razik 2014	Not reported	22	66% routine CT scan, 34% symptoms (not specified)	Not reported
Kim et al. 2020	Not reported	19	Not reported	PALN (38%), lung (25%), mediastinal LN (13%), supraclavicular LN (13%), para-oesophageal LN (13%), common iliac LN (13%), pelvis (13%)

### Risk of bias assessment

The NIH’s quality assessment tool for observational cohort and cross-sectional studies was utilised to assess the internal and external validity of the included studies. The overall quality was good for two studies and fair for the remaining three (Table 7). All studies lacked in the justification of sample size; however, this is reasonable as PALNM is a rare pathology, and all studies were retrospective in nature. Additionally, three studies did not adequately defined survival outcome measures [22, 24, 25], whilst one did not report the definition for PALNM [24].

**Table 7:** National Heart, Lung and Blood Institute’s (NIH) Quality Assessment Tool

Questions	Kim et al	Razik et al	Dumont et al	Min et al	Shibata et al
1. Was the research question or objective in this paper clearly stated?	Y	Y	Y	Y	Y
2. Was the study population clearly specified and defined?	Y	Y	Y	Y	Y
3. Was the participation rate of eligible persons at least 50%?	Y	Y	Y	Y	Y
4. Were all the subjects selected or recruited from the same or similar populations (including the same time period)? Were inclusion and exclusion criteria for being in the study prespecified and applied uniformly to all participants?	Y	Y	Y	Y	Y
5. Was a sample size justification, power description or variance and effect estimates provided?	N	N	N	N	N
6. For the analyses in this paper, were the exposure(s) of interest measured prior to the outcome(s) being measured?	Y	Y	Y	Y	Y
7. Was the timeframe sufficient so that one could reasonably expect to see an association between exposure and outcome if it existed?	Y (for short-term survival)	Y (for short-term survival)	Y	NR	Y
8. For exposures that can vary in amount or level, did the study examine different levels of the exposure as related to the outcome (e.g. categories of exposure, or exposure measured as continuous variable)?	NA	NA	NA	NA	NA
9. Were the exposure measures (independent variables) clearly defined, valid, reliable and implemented consistently across all study participants?	Y	Y	Y	Y	N (lacks definition of isolated RPR)
10. Was the exposure(s) assessed more than once over time?	NA (exposure = treatment for PALNM)	NA (exposure = PALNM and its treatment)	NA (exposure = PALNM and its treatment)	NA (exposure = PALNM and its treatment)	NA (exposure = PALNM and its treatment)
11. Were the outcome measures (dependent variables) clearly defined, valid, reliable and implemented consistently across all study participants?	Y	Y	N (no definition of survival measures)	N (no definition of survival measures)	N (no definition of survival measures)
12. Were the outcome assessors blinded to the exposure status of participants?	N (feasible for exposure, but not done as retrospective design)	N (feasible for exposure, but not done as retrospective design)	N (feasible for exposure, but not done as retrospective design)	N (feasible for exposure, but not done as retrospective design)	N (feasible for exposure, but not done as retrospective design)
13. Was loss to follow-up after baseline 20% or less?	Y	Y	Y	Y	Y
14. Were key potential confounding variables measured and adjusted statistically for their impact on the relationship between exposure(s) and outcome(s)?	Y	Y	N (only univariate analysis performed)	Y	Y
	Good	Good	Fair	Fair	Fair

## Discussion

Managing metachronous para-aortic lymph node metastases (m-PALNM) requires understanding of their prognostic significance, weighing up the risk of repeat resection and complications of surgery with overall survival, and comparing surgical management with systemic therapy. As isolated metachronous PALNM is a relatively rare pathology, there is no established treatment strategy. Synchronous resection of the primary tumour (s-PALNM) seems to be less controversial [11, 14, 16, 26–28]. Ichikawa et al*.* described an increased survival rate amongst patients having radical paraaortic lymph node resection at the time of primary resection compared to those having targeted lymphadenectomy [26]. Less is known about the survival benefit of resection of metachronous PALNM. The aim of this systematic review was to summarise the outcomes of surgical and non-surgical management of metachronous PALNM in colorectal cancer (CRC) to allow patients and clinicians to make informed decisions.

At the time of submission of this article, there were two very recently published review articles. Chen et al. reviewed both synchronous PALNM (s-PALNM) and m-PALNM articles [29]. Their m-PALNM articles included four of the studies also included in this article, except the study by Razik et al. [30] This article did not include a meta-analysis or risk-of-bias assessment and focused on examining survival outcomes and post-op complications. The authors were unable to make definitive recommendations and concluded that a prospective, multicentre study would be required to fully delineate the oncological outcomes of PALND. Wang et al. similarly reviewed both s-PALNM and m-PALNM articles with a focus on overall survival (OS) and post-operative complications [31]. The authors performed a meta-analysis for survival and reported that there was a 5-year OS benefit for s-PALNM and m-PALNM resection compared to non-operative treatment. This article concluded that although PALND (synchronous or metachronous) conferred survival benefits, there is significant paucity in the available literature on PALND and large randomised trials are warranted to confirm the benefits of lymphadenectomy.

This present review is not free from limitations. The number of studies extracted from the literature search is rather small, and the overall study period ranges between 1988 and 2014. Treatment strategies have changed for both operative and non-operative management of metastatic colorectal cancer within this period. Surgical strategies for managing metastases from CRC have been more aggressive during the past two decades, and chemotherapy guidelines have changed not only in terms of timing and duration of treatment but also in terms of the recommended regimens, especially after the broad utilisation of biological agents [32]. Secondly, the included studies represent small cohorts of patients, and subsequently the evidence derived from their results is rather weak. Pooling and meta-analysing the available data in order to provide a more robust conclusion from this systematic review was not feasible due to the heterogeneity in reporting of the survival outcomes. We acknowledge that the exclusion of studies not published in the English language will have contributed to this small sample size. Also, there was significant variability in the studies with regard to reports on resection margins and the use of neoadjuvant/ adjuvant chemotherapy and radiation, which makes the results difficult to generalise.

Despite the above limitations it is worth highlighting that resection of m-PALMN is reported to be a safe operation despite the fact that patients with recurrent disease undergo a second surgical procedure in a relatively short period after their colectomy. According to older reports, surgical resection for m-PALNM was thought to carry a high risk of complications [15], but this systematic review shows a 40% risk of complications of all grades and a low 30-day mortality rate of 0.8%. Given the fact that resection of m-PALMN has been reported to be open surgery in 100% of the cases, these morbidity and mortality rates should be considered reasonable.

Most importantly, this systematic review has demonstrated a significantly improved overall survival in patients undergoing surgical resection for m-PALNM compared to non-operative strategies. This is supported by three out of the five included studies, which compared survival outcomes between surgical and non-surgical management. Clear resection margins were reported in 88% of the cases, which is in keeping with the overall survival benefit observed after surgical management of m-PALNM.

In conclusion, the available evidence, as reported by this systematic review, suggests that surgical management of m-PALNM should be considered a safe procedure aiming to prolong survival. Based on the so far reported outcomes, a future randomised trial would be a safe and ethical step forward to provide more evidence in this field, ideally in a multicentre setting due to the low incidence of m-PALNM. In the era of multimodality treatment for metastatic colorectal cancer, surgical management of isolated metachronous lymph node metastases definitely represents a considerable option in carefully selected patients.

## Data Availability

The data used in this study is publicly available.

## Appendix 1

Centre for Evidence-Based Medicine’s criteria for levels of evidence for therapeutic studies.

**Table:**

Level	Evidence
1a	Systematic review (with homogeneity) of randomised controlled trials (RCT)
1b	Individual randomised controlled trial (with narrow confidence interval)
1c	All or none
2a	Systematic review (with homogeneity) of cohort studies
2b	Individual cohort study (including low quality RCT; e.g. < 80% follow-up)
2c	“Outcomes” research; ecological studies
3a	Systematic review (with homogeneity) of case–control studies
3b	Individual case–control study
4	Case-series (and poor quality cohort and case–control studies)
5	Expert opinion without explicit critical appraisal, or based on physiology, bench research or “first principles”

## Appendix 2

Preferred reporting items for systematic reviews (PRISMA) flow chart.
